# Traumatic events, daily stressors and posttraumatic stress in unaccompanied young refugees during their flight: a longitudinal cross-country study

**DOI:** 10.1186/s13034-022-00461-2

**Published:** 2022-03-31

**Authors:** Elisa Pfeiffer, Malte Behrendt, Sarah Adeyinka, Ines Devlieger, Marina Rota, Océane Uzureau, Floor Verhaeghe, Ine Lietaert, Ilse Derluyn

**Affiliations:** 1 Clinic for Child and Adolescent Psychiatry/Psychotherapy, Ulm University, Steinhoevelstraße 1, 89075 Ulm, Germany; 2 Department of Social Work and Social Pedagogy, Centre for the Social Study of Migration and Refugees, Faculty of Psychology and Educational Sciences, Ghent University, Henri Dunantlaan 2, 9000 Ghent, Belgium; 3 The United Nations University Institute On Comparative Regional Integration Studies (UNU-CRIS), Potterierei 72, 8000 Brugge, Belgium

**Keywords:** Unaccompanied young refugee, Youth, Trauma, PTSS, Flight, Daily stressor

## Abstract

**Background:**

Unaccompanied young refugees constitute an especially vulnerable population, reporting high rates of trauma and mental health problems. There is a significant gap in the literature on trauma and posttraumatic stress symptoms (PTSS) in unaccompanied young refugees who are still on the move and live in precarious circumstances such as refugee camps. This study therefore aimed to contribute to this gap by investigating pre- and peri-migration (potentially) traumatic experiences of unaccompanied young refugees; longitudinal trajectories of trauma, daily stressors and PTSS; and the impact of gender, trauma, and daily stressors on PTSS over time.

**Methods:**

This longitudinal, mixed-method, and multi-country study was conducted in various settings (e.g. refugee camps, reception centers) across nine European countries. A heterogeneous sample of *N* = 187 unaccompanied young refugees (78.4% male) from 29 different countries was assessed via interviews at 3 time-points during a period of 27 months. Data was analyzed via growth curve modelling.

**Results:**

Prevalence rates of (potentially) traumatic experiences ranged from 29.5 to 91.9%. Peri-migration traumatization remained stable over time (*b* = − 0.02; *p* = 0.371), but the number of reported daily stressors (*b* = − 0.24; *p* = 0.001) and PTSS scores significantly decreased over time (*b* = − 0.98; *p* = 0.004). Females reported higher PTSS compared with males at baseline (*p* = 0.002), but gender did not influence the longitudinal trajectory of PTSS. The pre-migration trauma load and daily stressors at baseline did not have a significant effect on PTSS at baseline or on the longitudinal trajectory.

**Conclusions:**

This is the first study to document not only the high numbers of traumatic events for unaccompanied young refugees pre- and peri- migration, but also the continued traumatization during flight, as well as high rates of daily stressors and PTSS. Humanitarian and political assistance is urgently needed to curb the often life-threatening conditions unaccompanied young refugees face during migration.

**Supplementary Information:**

The online version contains supplementary material available at 10.1186/s13034-022-00461-2.

## Background

Between 2017 and 2020 alone, more than 275 million people across the globe had been forcibly displaced for reasons ranging from armed conflict and persecution to economic pressure and natural disasters [1]; estimates further suggest that 30–43% of all refugees worldwide are below 18 years old [2]. Unaccompanied young people resettling as refugees, who are not protected by a parent or guardian but instead travel alone, constitute an especially vulnerable population. These young people face the atrocities of conflict-related violence and the numerous hardships of flight during crucial phases of their physical, emotional, social, and cognitive development [3]. However, because most studies investigating trauma and stressors during flight are conducted post-migration with young refugees resettling in a handful of Northern European countries [4–7], there is a significant lack of prospective epidemiological quantitative and longitudinal evidence on these young peoples’ specific traumatic experiences pre- and peri-migration.

Recent reviews and meta-analyses of mental disorders among unaccompanied young refugees revealed that 19–53% of this population reports posttraumatic stress symptoms (PTSS), while 10–33% report depression and 9–32% note anxiety [8, 9]; these are significantly higher prevalence rates compared to accompanied refugees and European-born minors [10]. The few existing longitudinal studies suggest a chronic trajectory of mental health problems in refugee minors [6, 11], although trauma-related mental health problems do not result from a single cause or stressor, but rather from complex causal chains [12]. Among several individual risk factors, such as being female [3, 11], there is strong evidence to suggest that cumulative exposure to traumatic events (often referred to as “trauma load”) is associated with subsequent psychological disturbances, such as PTSS, in this population [11]. However, in their longitudinal study with re-settled refugee minors in Belgium, Vervliet et al. [6] found that after trauma load, daily stressors, such as insufficient medical care or social support, have a significant impact on mental health. Again, these known risk factors for the development of PTSS have not been investigated with (unaccompanied) young refugees who are still on the move.

During their travels, unaccompanied young refugees often face harsh conditions, forced detention, and violence in transit countries. The effects of the transit conditions on unaccompanied young refugees are still relatively unknown, due to several practical difficulties linked with conducting research on migrants during their journey, are generally still relatively unknown. Refugees who want to reach Northern Europe usually cross the Mediterranean sea and enter the European Union via Spain, Italy, Greece, or Malta [13], but research on (mental) health and experiences in these transit countries is limited. Preliminary studies of transiting (adult) refugees in Greece [14] or Italy [15] report high trauma load and prevalence rates of mental health disorders, including posttraumatic stress disorder (PTSD), depression, substance abuse, and anxiety. Yet, none of these studies investigated unaccompanied young refugees in particular. Moreover, most studies report prevalence rates in single countries and lack comparisons between different settlement and transit countries.

Regarding the study setting, the growing body of research on mental health of unaccompanied young refugees has several crucial limitations, which substantially hamper the field’s understanding of unaccompanied young refugees' health in the context of migration. At present, very few studies have been conducted with unaccompanied young refugees living in refugee camps or detention centers, but have instead taken place in asylum centers [16], psychiatric hospitals [5, 17], or in the context of child welfare programs [18]. However, daily life in refugee camps and detention centers along country borders constitutes an especially challenging situation with a high number of daily stressors as there is often minimal access to shelter, food, water, education, or privacy. In sum, there has been no systematic research to fully capture the traumatic experiences and PTSS in unaccompanied young refugees during flight. The discrepancy between the importance of the topic and the dearth of data is striking.

This longitudinal follow-up study therefore aimed to fill the gap by focusing on unaccompanied young refugees living in a variety of settings (refugee camps, detention centers, housing units, informal settings), in different cities and regions across nine European countries, and over a time period of almost two years.

## Aims of this study

This study aimed at (A) investigating traumatic experiences reported by unaccompanied young refugees in transit at different time points of their migration, (B) to investigate longitudinal trajectories of trauma, daily stressors and PTSS symptoms in unaccompanied young refugees on the move, and (C) to assess the impact of gender, (potential) traumatic experiences, and daily stressors on PTSS in unaccompanied young refugees while in flight.

## Methods

This study was part of the CHILDMOVE project, a European Research Council [HORIZON project number: 714222] funded mixed-method multi-site and multi-country (Belgium, Italy, Greece, Libya). The CHILDMOVE project aimed to increase knowledge about the impact of experiences occurring during the flight on the psychological wellbeing of unaccompanied young refugees in relation to the impact of past traumatic experiences in their home countries and to daily stressors in their current countries of stay.

### Participants

In three out of the four samples, we only included young refugees with a self-declared age of under 18, but given the difficulties related to age and age assessment in the policy related to unaccompanied refugee minors [19], we decided to use a broader framing of unaccompanied young refugees, referring to ‘children, adolescents and young adults who are separated from their parents and are thus migrating on their own and not anymore cared for by an adult who is by law or custom responsible for doing so’. Since the distinction between forced and voluntary migration is often unclear, and because these young people seldom make the decision to migrate by themselves [20], we use the term “refugee” inclusively for all young migrants who felt forced to flee. The study sample consisted at baseline of *N* = 187 adolescents and young adults (*M*_*age*_ = 16.78; *SD*_*age*_ = 2.36; 21.6% female) with *n* = 64 (34.2%) in Italy, *n* = 79 (42.2%) in Belgium and *n* = 44 (23.5%) in Greece. Demographic information of the entire study sample and subsamples per country is presented in Table 1.

**Table 1:** Sociodemographic characteristics of participants across country at baseline (M1)

	Subsamples			
	Italy n = 62	Belgium n = 79	Greece n = 42	Total n = 187
Gender				
Male n (%)	32 (50.8)	72 (91.1)	41 (95.3)	145 (78.4)
Female n (%)	31 (49.2)	7 (8.9)	2 (4.7)	40 (21.6)
Age M (SD); range	17.97 (3.2); 13–25	15.87 (1.2); 13–20	16.23 (1.2); 13–19	16.78 (2.4); 13–25
Country of origin n (%)*				
Central Africa^A^	1 (1.6)	9 (11.4)	–	10 (5.5)
West Africa^B^	39 (62.9)	13 (16.5)	1 (2.4)	53 (29.0)
East Africa^C^	13 (21.0)	16 (20.3)	–	29 (15.8)
North Africa^D^	9 (14.5)	4 (5.1)	1 (2.4)	14 (7.7)
Middle East^E^	–	9 (11.4)	9 (21.4)	18 (9.8)
South Asia^F^	–	22 (27.8)	31 (73.8)	53 (29.0)
Southeast Europe^G^	–	6 (7.6)	–	6 (3.3)
Time since left home country (in months) M, SD (range)	16.66, 9.38 (2–49)	15.5, 13.56 (0–72)	17.75, 1.30 (2–48)	16.39, 12.15 (0–72)
Contact with parents				
Yes n (%)	31 (77.5)	23 (35.9)	25 (65.5)	79 (55.6)
Living situation n (%)				
Reception center for minors only	11 (17.2)	79 (100)	–	90 (48.4)
Collective reception center for adults and minors—no separation between adults and minors	2 (3.1)	–	–	2 (1.1)
Collective reception center for adults and minors—minors separated from adults	16 (25)	–	–	16 (8.6)
Detention center	–	–	12 (27.9)	12 (6.5)
House, studio or flat	26 (40.6)	–	–	26 (14)
Informal camp	9 (14.1)	–	–	9 (4.8)
Shelter for unaccompanied minors	–	–	31 (72.1)	31 (16.7)
Asylum status n (%)				
Procedure in process	12 (18.8)	79 (100)	22 (53.7)	113 (61.4)
Granted family reunification -waiting for transfer	2 (3.1)	–	2 (4.9)	4 (12.5)
Temporary documents-less than 1 year	23 (35.9)	–	–	23 (12.5)
Temporary documents-less than 2 years	1 (1.6)	–	–	1 (0.5)
Definite residence permit	3 (4.7)	–	2 (4.9)	5 (2.7)
Negative decision on procedure-no documents	–	–	3 (7.3)	3 (1.6)
Never applied for a procedure-no documents	23 (35.9)	–	12 (29.3)	35 (19)

A, Cameroon, Chad, Congo, Dem. Rep., Gabon; B, Cote d’Ivoire, The Gambia, Guinea, Mali, Nigeria, Senegal, Sierra Leone; C, Eritrea, Ethiopia, Somalia, South Sudan; D, Algeria, Egypt, Libya, Morocco, Sudan, Tunesia; E, Iran, Iraq, Lebanon, Syria; F, Afghanistan, Pakistan; G, Albania, Serbia

^*^n, 4 missing data

### Recruitment, procedure, and setting

In order to reflect the diversity of settings through which unaccompanied young refugees’ move, we recruited participants both in transit/first arrival countries (Greece, Italy) and in a settlement country (Belgium). Greece and Italy in particular were selected as main entry points in Europe at the time of the start of the study (2017). In Greece, the recruitments and first assessments were conducted in centers for unaccompanied young refugees, a detention center, and a refugee camp (Reception and Identification Centre), in Thessaloniki, Samos, and Athens. The sample of participants recruited in Italy is a combination of two study samples: one sample of participants was recruited from three different centers for refugees and victims of trafficking in Campagnia, Piemonte, and Sicily. The other sample of participants was recruited in a first reception center located in Sicily, as well as in transit camps and informal settlements in Lazio and Imperia regions. The recruitment and first assessments in Belgium were conducted in the Brussels region at two reception centers for newly arrived unaccompanied young refugees. When feasible, the researchers engaged in participant observation and spent time with the young people to build rapport before asking them whether they would agree to participate. Based on information concerning the socio-demographic characteristics of the group of unaccompanied refugee minors in the country of recruitment, as provided by national authorities, we aimed to make the samples representative in each of these countries in terms of gender and nationality to reflect the demographic composition of unaccompanied young refugees in each region.

Participants were assessed at three measurement time points: Baseline (M1), a follow-up (M2) after approximately 8 months (*M* = 8.0, *SD* = 2.4, range = 5–17), and another follow-up (M3) after approximately 21 months (*M* = 20.5, *SD* = 3.0, range = 12–27). At M2, *n* = 66 (35.3%) dropped out, and at M3 altogether *n* = 102 (54.5%) had dropped out. For the numbers of missing data please see Additional file 1. After M1, the researchers invited the participants to exchange contact details and offered to stay in touch with the participants via phone calls, messages or social media. Via these contact details, participants were invited to attend follow-up assessments, and researchers travelled to their current location. Follow-up assessments took place in Greece, Italy, Belgium, Germany, the Netherlands, the United Kingdom, Switzerland, Spain, and Malta. All interviews were conducted between October 2017 and October 2020. All of the measures used, as detailed below, were translated into 13 languages and were administered by trained assessors (authors MB, SA, OU, MR). The semi-structured interviews, which were also used to produce qualitative data, allowed assessors to ask follow-up questions to the participants’ responses, such as how the participants coped with the different symptoms, and what helped them (e.g., in terms of social support or care structures) in dealing with the stressors and symptoms. Interpreters and cultural mediators were employed if the bi-/multi-lingual researcher and the participant did not speak a common language or due to participant preference.

### Ethics committee approval

All participants and, in the case of minors, if already appointed, their legal guardians, gave their informed written consent before being enrolled in the study. The study protocol was approved by the institutional review board (IRB) at Ghent University (#2017-23-Ine Lietaert) and national ethical bodies in Greece and Italy.

## Measures

### Choice of primary measure

The “Reactions of Adolescents to Traumatic Stress questionnaire (RATS)” (19) was chosen as the primary measure for this study, as PTSD is the most prevalent reported mental health disorder in unaccompanied young refugees. The RATS is a multicultural self-report measure assessing the prevalence of PTSS according to DSM-IV criteria. The measure has been widely used with migrant and refugee youth, including unaccompanied minors. For use with refugee and migrant youth, the measure has been translated and back-translated into 19 different languages. The items range from “not at all” (1) to “always” (4) (possible range: 10–40) and Cronbach’s alpha was acceptable to good (M1: α = 0.76; M2: α = 0.81; M3: α = 0.84). In this study, we implemented a short version of the RATS in order to keep the assessment brief for ethical reasons. There is no information on clinical cut-offs and clinically significant change yet, as this is a short version of the measure (10 instead of 22 items). The measure is available free of charge from the authors, upon request.

### Traumatic experiences

The “Stressful Life Events questionnaire (SLE)” (21) is a self-report measure assessing 10 different potentially traumatic events at three time points: pre-migration, peri-migration and since arrival in the current host country. The measure has been widely used in samples of (unaccompanied) refugee adolescents [10, 22].

### Daily stressors

The “Daily Stressors Scale for Young Refugees (DSSYR)” (23) is a self-report measure consisting of 20 potential daily stressors (and one open question for other daily stressors) and assessing to what extent these have been experienced during the past month on a 4-point Likert scale ranging from “never” (1) to “always” (4) (possible range: 20–80). The questionnaire was developed on the basis of the Columbia Impairment Scale (CIS), the Adolescents Complex Daily Stressors Scale (ACDSS), and the authors’ own experiences in the field. Although this questionnaire is widely used in research with unaccompanied young refugees, the validation study of this measure has not yet been published.

### Statistical analysis

Descriptive statistics were conducted (using SPSS 26 and R Studio) to outline socio-demographic characteristics, the prevalence of traumatic events (pre- and peri-migration), daily stressors, and PTSS (M1-M3). Sum scores of traumatic experiences (pre- and peri-migration; SLE), PTSS (RATS), and daily stressors (DSSYR) were conducted by calculating mean scores and multiplying the mean scores with the number of items, to also receive a sum score of participants who have missing items. For the peri-migration trauma load score, the different time points “on my way here” and “since arrival to this country” were combined. Data was missing at random and PTSS scores of previous time points did not predict later attrition, neither at M2 (*R*^2^ = 0.002; *b* = 0.003; *p* = 0.589) nor at M3 (*R*^*2*^ = 0.01; *b* = 0.001; *p* = 0.912). Missing data was replaced by employing multiple imputation with five imputed datasets. The data were then analyzed via growth curve modelling (GCM) [24] to estimate inter-individual variability in intra-individual patterns of change over time, as GCM can handle missing data and unequally spaced time points, as well as time-varying co-variates, particularly well. Because the data was not normally distributed, we used robust standard errors and chi-squared statistics for correction. We applied a structural equation model (SEM) framework to the models. In the first unconditional model, we included the three dependent time-variant variables (PTSS, peri-migration trauma load, daily stressors) to investigate the longitudinal trajectories of the three concepts over time. In the second model with only PTSS as the outcome variable, we added the time invariant factors gender, pre-migration trauma (M1), and daily stressors (M1) as potential predictors of the outcome over time and used the study country as a control variable. For all GCM analysis, *n* = 3 participants were not included due to missing RATS scores at M1. The GCM analyses were run with the R packages lavaan 0.6–8.1604 (25) and semTools 0.5–3.910 (26).

## Results

### Traumatic experiences

The different potentially traumatic experiences per subsample (study country) and total study sample are depicted in Table 2. There were high prevalence rates of potentially traumatic experiences across all samples, ranging from 29.5–91.9%. The experiences most often reported across samples were witnessing (*n* = 171; 91.9%) and experiencing (*n* = 158; 84.9%) physical violence, as well as other stressful events with great danger (*n* = 165; 91.2%). The most common events in the home country were drastic changes in the family (*n* = 120; 64.8%) and other very stressful life events with great danger (*n* = 83; 45.9%). The most frequent events for “on the way to this country” were witnessing physical violence (*n* = 121; 65%) and other stressful life event with great danger (*n* = 114; 63.1%). Lastly, the most often reported events since arrival to the current host country were the experience of physical violence (*n* = 30; 16.1%), drastic changes in the family (*n* = 27; 14.5%), and detention/imprisonment (*n* = 27; 14.5%). Among the least frequently reported events were sexual violence (*n* = 54; 29.5%) and forced separation from family (*n* = 90; 48.9%). Notably, the sample in Italy reported considerably more sexual violence (46.9%) compared with the other samples (17.3–25%); however, this can be explained by the inclusion in the Italian sample of a small subsample of Nigerian females who had experienced sex trafficking. The range in all categories of reported potentially traumatic events was similarly high across study countries, although the sample in Italy reported the highest prevalence rates in eight events, compared with three events in the sample in Belgium and none in the sample in Greece.

**Table 2:** Potential traumatic experiences/stressful life events across samples and location of event at baseline (M1)

	Subsamples			Total sample
	Italy	Belgium	Greece	
Drastic changes in family, n (%)				
Not experienced	23 (35.9)	8 (10.4)	7 (15.9)	38 (20.5)
In my home country	27 (42.1)	62 (80.5)	31 (70.4)	120 (64.8)
On my way to this country	6 (9.4)	8 (10.4)	10 (22.7)	24 (12.9)
Since arrival in this country/place	13 (20.3)	3 (3.9)	11 (25)	27 (14.5)
Total	41 (64.1)	69 (89.6)	37 (84.1)	147 (79.5)
Forced separation from family, n (%)				
Not experienced	46 (71.9)	22 (28.9)	26 (59.1)	94 (51.1)
In my home country	10 (15.7)	48 (63.1)	13 (29.5)	71 (38.5)
On my way to this country	9 (14.1)	9 (11.8)	3 (6.8)	21 (11.3)
Since arrival in this country/place	1 (1.6)	3 (3.9)	4 (9)	8 (4.3)
Total	18 (28.1)	54 (71.1)	18 (40.9)	90 (48.9)
War or armed military conflict, n (%)				
Not experienced	20 (31.1)	26 (33.3)	19 (43.2)	65 (34.9)
In my home country	15 (23.4)	46 (59)	23 (52.3)	84 (45.2)
On my way to this country	37 (57.8)	8 (10.3)	9 (20.4)	54 (29.1)
Since arrival in this country/place	–	–	3 (6.8)	3 (1.6)
Total	44 (68.8)	52 (66.7)	25 (56.8)	121 (65.1)
Forced to work, n (%)				
Not experienced	21 (33.3)	30 (39)	24 (55.8)	75 (41)
In my home country	4 (6.4)	18 (23.4)	15 (34.9)	37 (20.1)
On my way to this country	31 (49.2)	30 (39)	5 (11.6)	66 (36)
Since arrival in this country/place	11 (17.5)	2 (2.6)	3 (7)	16 (8.7)
Total	42 (66.7)	47 (61)	19 (44.2)	108 (59)
Experienced physical violence, n (%)				
Not experienced	7 (11.1)	13 (16.5)	8 (18.2)	28 (15.1)
In my home country	14 (22.2)	34 (43.14)	23 (52.2)	71 (38.2)
On my way to this country	44 (69.8)	48 (60.8)	21 (47.7)	113 (60.7)
Since arrival in this country/place	15 (23.7)	1 (1.3)	14 (31.8)	30 (16.1)
Total	56 (88.9)	66 (83.5)	36 (81.8)	158 (84.9)
Witnessed physical violence, n (%)				
Not experienced	3 (4.7)	6 (7.7)	6 (13.6)	15 (8.1)
In my home country	14 (21.8)	45 (57.7)	24 (54.4)	83 (44.7)
On my way to this country	54 (83.4)	45 (57.7)	22 (49.9)	121 (65)
Since arrival in this country/place	5 (7.8)	–	16 (36.3)	21 (11.3)
Total	61 (95.3)	72 (92.3)	38 (86.4)	171 (91.9)
Experienced sexual violence, n (%)				
Not experienced	34 (53.1)	62 (82.7)	33 (75)	129 (70.5)
In my home country	4 (6.3)	5 (6.6)	6 (13.6)	15 (8.1)
On my way to this country	26 (40.6)	9 (12)	4 (9.1)	39 (21.2)
Since arrival in this country/place	8 (12.5)	–	5 (11.4)	13 (7)
Total, n (%)	30 (46.9)	13 (17.3)	11 (25)	54 (29.5)
Detention or imprisonment, n (%)				
Not experienced	12 (18.8)	18 (23.1)	11 (25)	41 (22)
In my home country	9 (14)	18 (23.1)	7 (16)	34 (18.3)
On my way to this country	49 (76.5)	49 (62.8)	13 (29.6)	111 (59.7)
Since arrival in this country/place	6 (9.4)	3 (3.8)	18 (40.9)	27 (14.5)
Total	52 (81.3)	60 (76.9)	33 (75)	145 (78)
Other very stressful life event with great danger, n (%)				
Not experienced	7 (11.9)	4 (5.1)	5 (11.4)	16 (8.8)
In my home country	9 (15.3)	50 (64.1)	24 (54.6)	83 (45.9)
On my way to this country	45 (76.3)	42 (53.9)	27 (61.4)	114 (63.1)
Since arrival in this country/place	14 (23.8)	–	11 (25.1)	25 (13.9)
Total	52 (88.1)	74 (94.9)	39 (88.6)	165 (91.2)
Other very stressful life event with someone else in great danger, n (%)				
Not experienced	10 (16.4)	13 (16.7)	8 (18.6)	31 (17)
In my home country	7 (11.5)	38 (48.7)	19 (44.3)	64 (35.2)
On my way to this country	45 (73.8)	36 (46.1)	23 (53.6)	104 (57.1)
Since arrival in this country/place	5 (8.2)	1 (1.3)	14 (32.7)	20 (10.9)
Total	51 (83.6)	65 (83.3)	35 (81.4)	151 (83)

Total means number of participants who report the event independent of location. The % value is valid percent for each subsample and time point

### Unconditional growth models

**Fig. 1:**
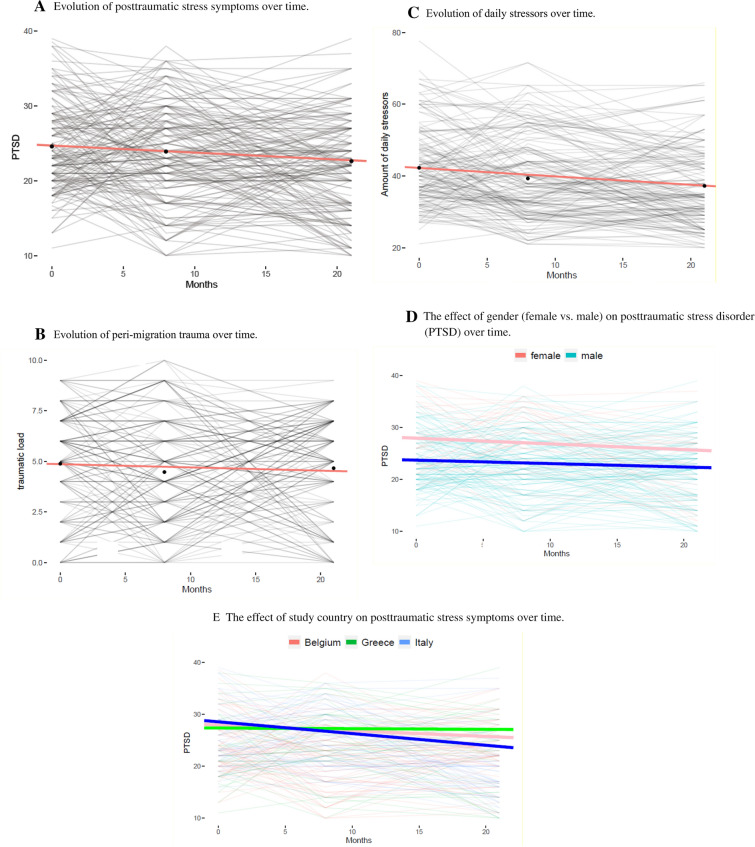
**A** Evolution of posttraumatic stress symptoms over time. **B** Evolution of peri-migration trauma over time. **C** Evolution of daily stressors over time. **D** The effect of gender (female vs. male) on posttraumatic stress disorder (PTSD) over time. **E** The effect of study country on posttraumatic stress symptoms over time

Table 3 presents the means, standard deviations, and range of pre- and peri-migration trauma, PTSS, and daily stressors. The models provided good model fit for PTSS (χ^2^(1) = 0.21, *p* = 0.714, *CFI* = 1.0, *SRMR* = 0.0080, *RMSEA* = 0.000), peri-migration trauma (χ^2^(1) = 1.72, *p* = 0.223, *CFI* = 0.99125, *SRMR* = 0.02900, *RMSEA* = 0.05100), and daily stressors (χ^2^(1) = 2.4098, *p* = 0.223, *CFI* = 0.9816, *SRMR* = 0.0264, *RMSEA* = 0.0718). For results of the unconditional growth models on peri-migration trauma, PTSS, and daily stressors, please see Table 4 and Fig. 1A–C. PTSS scores significantly decreased over time and there was a marginally significant variance at M1 between the individuals, meaning that marginal significant differences between the respondents were present at M1 (*t*(72,776) = 1.98; *p* = 0.052). The evolution over time also did not differ significantly between the individuals (*t*(7,411) = 1.17; *p* = 0.277) and the covariance between intercept and slope (β = − 0.08, *SE* = 0.35) was not significant (*t*(11.197) = − 0.23; *p* = 0.821). The peri-migration trauma score was stable over time, with a significant variance at M1 between the individuals (*t*(31,040) = 3.77; *p* < 0.001), but the evolution over time also did not differ significantly between the individuals (*t*(6,680) = 216; *p* = 0.835). The covariance between intercept and slope (β = − 0.17, *SE* = 0.09) was not significant (*t*(15,678) = 15.68; *p* = 0.077), meaning that the intercept did not affect the slope. The daily stressor score significantly decreased over time. The variance varied significantly between individuals at M1 (*t*(35,272) = 3.91; *p* < 0.001), but the variance in the slope varied between individuals (*t*(17,25) = 2.28; *p* = 0.036) and the covariance between intercept and slope (β = − 2.04, *SE* = 0.99) was not significant (*t*(17,41) = − 2.05; *p* = 0.056).

**Table 3:** Descriptive data on pre- and peri-migration trauma, daily stressors, and PTSS across all measurement time points (M1–M3) (N = 187)

	M1 M (SD), range	M2 M (SD), range	M3 M (SD), range
Pre-migration trauma	3.43 (2.50), 0–9	n/a	n/a
Peri-migration trauma*	5.98 (3.04), 0–14	5.98 (3.68), 0–15	6.50 (2.85), 0–12
Daily stressors	41.72 (9.00), 21–78	38.58 (9.37), 21–72	37.05 (10.80), 20–66
PTSS symptoms	24.55 (5.91), 11–39	23.92 (6.44), 10–38	22.38 (6.37), 10–39

n/a indicates no data available. Since all participants were on the move during the study, traumatic events prior migration (pre-migration) are only reported at baseline

*M1* baseline, *M2* 6–12 month follow-up, *M3* 18–24 month follow-up, *PTSS* posttraumatic stress symptoms

*peri-migration and host-country combined, see GCM model

**Table 4:** Unstandardized estimates and standard errors of means and variances or parameters of unconditional growth models

	Intercepts			Variance		
	Estimate	SE	p	Estimate	SE	p
PTSS intercept	24.61	0.61	< 0.001	12.03	6.09	0.052
PTSS slope	− 0.10	0.04	0.035	0.05	0.04	0.277
Peri-migration trauma intercept	4.85	0.32	< 0.001	5.34	1.42	0.001
Peri-migration trauma slope	− 0.02	0.02	0.371	0	0.01	0.835
Daily stressors intercept	42.00	1.06	< 0.001	79.21	20.25	< 0.001
Daily stressors slope	− 0.24	0.06	0.001	0.22	0.10	0.036

### Conditional growth curve model

The model with time-invariant predictors (gender, study country and pre-migration trauma) fit the data well: χ2(6) = 8.56750, *p* = 0.254, *CFI* = 0.97000, *SRMR* = 0.03175, *RMSEA* = 0.04675. Please see Table 5 and Fig. 1D–E for the results of the model. There was no significant effect of the control variable study country, neither at baseline nor on the longitudinal trajectory of PTSS. At baseline, females reported higher PTSS compared with male participants, but gender did not influence the longitudinal trajectory of PTSS over time. The pre-migration trauma load and daily stressors at M1 did not have a significant effect on either PTSS at baseline or the longitudinal trajectory.

**Table 5:** Standardized estimates of the time-invariant coefficients (study country, gender, peri-migration trauma (M1), daily stressors (M1)) and growth parameters of PTSS (n = 185).

	Intercept			Slope		
	Estimate	SE	p	Estimate	SE	p
Country (Greece)*	− 0.89	1.77	0.613	0.09	1.00	0.392
Country (Italy)*	1.05	1.51	0.490	− 0.16	0.10	0.106
Gender (male)**	− 4.98	1.58	0.002	0.07	0.11	0.560
Pre-migration trauma M1	0.53	0.29	0.069	− 0.02	0.02	0.248
Daily stressors M1	0.07	0.05	0.164	0.00	0.00	0.973

*reference group is Belgium

**reference group is female

## Discussion

This is the first study to investigate trauma, daily stressors, and PTSS in a cross-country sample longitudinally with unaccompanied young refugees who are currently on the move. The study results revealed an extremely high trauma load pre- and peri-migration across all unaccompanied young refugee subsamples. In particular, interpersonal trauma, such as witnessing and experiencing physical violence, was common across almost all unaccompanied young refugees reporting this at any time point during their flight, or in their home country. Alarmingly, even the least frequently occurring events (sexual violence and forced separation from family) were reported by at least one-third of the participants. The peri-migration trauma load also remained stable over time, demonstrating ongoing traumatization during flight. This study’s results contribute to the growing body of research on cumulative trauma in unaccompanied young refugees indicating a high and continuous trauma load in a heterogeneous sample of unaccompanied young refugees at different time points as they move through different study settings (e.g., refugee camps, detention, asylum centers), regions, and countries in Europe.

Contrary to the continuous traumatization, however, the number of reported daily stressors significantly decreased over time, which may be explained by the extraordinary resilience of young refugees, often in the form of social networks [27], which might encompass a potential adaptation process to the harsh conditions they are facing on their flight [28]. Another possible explanation is that most participants in our sample journeyed from transit countries, where conditions are often extremely harsh, to settlement countries in Northern Europe, where young refugees likely face fewer daily stressors.

Unlike other longitudinal studies of unaccompanied young refugees in Norway [11] and Belgium [6] showing that PTSS remained stable in the course of 6, 12, and 18 months post-arrival in the host country, this study’s results suggest a small but significant decrease of PTSS over time. A key distinguishing factor when comparing these studies, which were conducted mainly with refugee minors who had already arrived in their host country, with this study is that the longitudinal course of this study of unaccompanied young refugees took place against the backdrop being “on the move” in several different countries. Whereas settlement may provide more stability in the daily life and integration process, being in transit is associated with unpredictable changes in daily needs and stressors, as well as continuous traumatization; this may result in a spontaneous increase, but can also cause a decrease of symptoms due to natural symptom remission common in PTSD [29]. Moreover, in order to reach their next destination, refugees on the move are often in “survival mode,” shifting their focus away from internal symptoms, such as intrusive thoughts or difficulty concentrating, and instead toward external stressors. In line with numerous studies on risk factors for PTSS development in unaccompanied young refugees [3], this study also showed that female gender was associated with higher PTSS at baseline and with a similar longitudinal PTSD trajectory.

The results indicate that the pre-migration trauma load did not significantly predict the baseline and longitudinal PTSS score; this is contrary to the assumption of the dose–response model in which an individual’s risk of response varies with the magnitude of the stressors [30], which was found in numerous studies on unaccompanied young refugees PTSS levels [3]. Similarly, many studies have shown that daily stressors have a significant impact on PTSS in unaccompanied young refugees [11], but in our sample the daily stressors at baseline did not predict PTSS. A possible explanation for this finding could be that the generally high trauma and daily stressors load in all participants resulted in a ceiling effect. The cognitive model of trauma and PTSD by Ehlers and Clark [31] indicates that the subjective appraisal of the event and current situation (such as sense of threat) has a crucial impact on the development and chronification of symptoms; if so, traumatic events during flight and in the host country might have an important impact on PTSS longitudinally. Unfortunately, time-variant factors like potential trauma and daily stressors during flight and in the host country resulted in an insufficient model fit for our sample (χ2(17) = 88.6614, *p* =  < 0.001, *CFI* = 0.7220, *SRMR* = 0.0628, *RMSEA* = 0.1630) which is why results are not reported. In order to better understand the maintenance of PTSS during migration and settlement, we encourage fellow scholars whose studies included larger sample sizes to take not only time-invariant but also time-variant predictors into account.

## Limitations

Although this study has many strengths, such as the implementation of an interview format, the longitudinal study design and multi-country context, the translation of the questionnaires, and the presence of interpreters during assessment, there are several possible limitations to the generalizability of these findings. First, the study comprises a very heterogeneous and generally representative, but rather small, sample. Unfortunately, the response rate of invited participants who would end up participating in the study was not systematically assessed due to the naturalistic and youth-friendly approach in the recruitment processes. Second, some participants could be considered “settled” rather than being “on the move” as they intended to stay in the current country, particularly in the Belgian sample. Acknowledging that settlement is a subjectively and objectively complex process, subject to numerous definitions within the relevant literature, we chose to refer to the entire sample as “on the move” to reflect the dynamic migration context. Moreover, all participants had in fact recently been in transit and *n* = 17 (21.5%) of the Belgium sample left Belgium after M1. Third, although the measure DSSYR has been widely used in the field of refugee mental health (11), it might not fully capture the diversified stressors faced by the participants in each setting, and the measure is still not validated in its current form. However, a recent validation study by the authors is in preparation for submission. Fourth, because daily stressors and traumatic events were assessed via checklists and treated as sum scores in the analysis, which is a common approach in the field, potentially important characteristics, such as the intensity, frequency or duration of events, were neglected and thus it is possible that there may be some bias or oversimplification of the results. Moreover, while the SLE checklist does include several refugee-specific pre- and peri-migration events, such as imprisonment or war-related events, one measure cannot capture all of the potentially traumatic events experienced by the participants; for example, categories such as terrorism or torture are not on the checklist. Fifth, due to ethical considerations that required keeping the assessment short and focusing on the narrative of the person, we used a shorter version of RATS, which made it difficult to compare results with other studies using RATS and draw conclusions on clinical significance. Sixth, this study focused on several specific risk factors and their relation to PTSS, and thus neglects other potential risk factors and trauma-related mental health areas that are relevant for unaccompanied young refugees. Future studies should aim to replicate these findings in unaccompanied young refugees on the move and further investigate known protective and risk factors, such as social support, that have implications the development of mental health issues, but such studies should also seek to deepen the understanding of how aspects such as resiliency, function, and quality of life may have important consequences as well. Seventh, as the majority of the study participants were male, findings on gender effects should be interpreted with caution.

## Conclusion

The results of this longitudinal multi-site and multi-country study provide an important contribution to the current literature on the mental health of unaccompanied young refugees, shedding light on continuous traumatization, high rates of reported daily stressors, and PTSS in young refugees who are still on the move. Humanitarian assistance and well-being centered political intervention is urgently needed to curb the often inhumane and threatening conditions unaccompanied young refugees face during their journey. Structured protection, medical, and psychological support in particular are crucial for this population, who are on the move during an important phase of their emotional, cognitive, and social development but lack known protective factors, such as social support and family guidance. It is critical that policymakers prioritize actions aimed at reinforcing the child protection system along all migratory routes.

## Supplementary Information

**Additional file 1.** Number of missing data for each measurement and time point.

## Data Availability

For privacy reasons the data is not fully available online. Proposals should be directed to ilse.derluyn@ugent.be; to gain access, data requestors will need to sign a data access agreement.
