# Supplementary material for: Traumatic events, daily stressors and posttraumatic stress in unaccompanied young refugees during their flight: a longitudinal cross-country study
Source: Child Adolesc Psychiatry Ment Health. 2022 Mar 31;16:26. doi: 10.1186/s13034-022-00461-2 (PMC8974188; doi:10.1186/s13034-022-00461-2)
Supplement: Supplementary file 1 — Additional file 1. Number of missing data for each measurement and time point. [file 13034_2022_461_MOESM1_ESM.docx]

| **Supplementary Information**  **Additional File 1.** Number of missing data for each measurement and time point | | | |  |  |
| --- | --- | --- | --- | --- | --- |
|  | PTSD  n (%) | Trauma*  n (%) | Daily stressors  n (%) | | |
| M1 | 0 | 0 | 19 (10.20%) | | |
| M2 | 68 (36.36%) | 73 (39%) | 67 (35.82%) | | |
| M3 | 107 (57.22%) | 106 (56.68%) | 106 (56.68%) | | |
| *trauma includes pre-migration, peri-migration trauma and trauma in the host country as this was one questionnaire. | | | | | |
